# Supplementary material for: Patterns of Intron Gain and Loss in Fungi
Source: PLoS Biol. 2004 Nov 30;2(12):e422. doi: 10.1371/journal.pbio.0020422 (PMC532390; doi:10.1371/journal.pbio.0020422)
Supplement: Table S1 — Also available at http://genes.mit.edu/NielsenEtAl/. (4.3 MB ZIP). [file pbio.0020422.st001.zip › NielsenEtAl/html/901.html]

AN7044.1.NCU06974.1.MG06040.1.FG05635.1


```
 CLUSTAL W (1.82) Multiple Sequence Alignments - Introns Inserted


Sequence 1: NCU06974.1	316 aa
Sequence 2: MG06040.1	329 aa
Sequence 3: FG05635.1	309 aa
Sequence 4: AN7044.1	361 aa
Alignment Length: 390 aa
Number Identitical Residues: 98 aa
Alignment Score (without introns) 5871


MG06040.1 	------------~---------------------------------------MHS~HSGQ
NCU06974.1	------------~-----------------------------------MAFTMHS~HSGQ
FG05635.1 	------------~-----------------------------------MAFTMHS~HSGE
AN7044.1  	MLLPKQMFIYAI2YPTNSVDYHAVVLISLISPPLHPIYVQFLISGGASYPIALVL2HSGQ
          	   ...    :   .:.: .  :    :  :..  .   .   :..:: . ::   ***:

MG06040.1 	FCPGHAKDQLEDVILHAISIGYKTMGLSEHMPRTQLCDLYPEE0LVPDP-HASLAELMPR
NCU06974.1	FCPGHAKDQLEEVIRHAISVGYRTIGLTEHMPRTQLSDLYPEE0LDPDQGQTTLAQLVPR
FG05635.1 	FCPGHAKDQLEEIVKQAVSIGYKTIGFTEHMPRYDLRDLYPEE0LENPQ--AALEALPPR
AN7044.1  	FCPGHAKDSLEEVIQLAIAKNFQVFCLTEHMPRGEQ-DYYPEE0IEANQ---TKAWMIAN
          	********.**:::  *:: .::.: ::***** :  * **** :       :   : ..

MG06040.1 	HAAYMTEARRLQKKYADRITLLIGFEGEFIR-SEYGTLVRSLADGNGDPSYFQNGDSKLV
NCU06974.1	HASYLAEAQRLQAKYASQIHILIGFEGEWIRGAEYGPLISS-----------------LL
FG05635.1 	HESYLVEAQRLRHKYASQIHILIGFEAEFIR-SSCATHVRR------------------L
AN7044.1  	EANYFREATRLREKYADQIKILIGFECDWIR-PESRTLVET------------------S
          	.  *: ** **: ***.:* :***** ::** ..  . :                     

MG06040.1 	TDAGKVDYFIGSLHHGAGGIPIDFDRATYLRSVEAAGPNGEEDLFVHYYDQQFEMLQALR
NCU06974.1	SSAPCVDYFIGSIHHTAG-IPIDFDKTMFLQAQDACG-GLEEKLWERYYDEQFEMLNATR
FG05635.1 	AEHPIVDYFIGSVHYVHN-MPIDYSKEIFLTAQGKSSQGTEESLYEDYYDLQYEMLTELK
AN7044.1  	MRAFPFEFFMGSIHHTKT-IPIDYDREMYEKARDLAG-GSDEKLFEAFFDEQLDMLKQLK
          	     .::*:**:*:    :***:.:  :  :   .. . :*.*:  ::* * :**   :

MG06040.1 	PPIVGHFDLIRLMSEEPGRNPSAWSPNRVWPLIKRNLAFVASYGGWLECNSSALRKGLAE
NCU06974.1	PKVVGHFDLIRLLSEEPDKRVKGWR-DGVWERMKRNLEVCKGLGAWLEVNTSALRKGLEE
FG05635.1 	PRVVGHFDLIRLMSEDPERDLRQWK--GAWDRVLRNLTLAKEQDGWLEVNSAALRKGLAE
AN7044.1  	PVVVGHFDLIRLKSDDPERSFKQWP--GVWERILRNLDYVAGYGGILELNSAALRKGMSE
          	* :********* *::* :    *    .*  : ***      .. ** *::*****: *

MG06040.1 	PYPCRPIAE~EWVRLGGKFTMSDDSHGIAQVATNYVRALDYLES-LGVNEVWTYDRAKEG
NCU06974.1	PYPGREVAE0EWIKMGGKFTFSDDSHGIAQVATNYTRGLDYLES-LGVTELWTLERVPHP
FG05635.1 	PYPGRQIAE0EWIKMGGKFTFSDDSHGIAQVATNYARTITYLES-LGVKEVWTLKRSSHT
AN7044.1  	PYPKAEICQ0EFLARGGRFCFSDDSHGLDQIGLNYARMLEFIETNTDIKTLYYLDLGNEP
          	***   :.: *::  **:* :******: *:. **.* : ::*:. .:. ::  .   . 

MG06040.1 	-------SELVEKGVSFTEFRGSLRLPTTASKTS
NCU06974.1	GTERDIKSELRDKAVTIFEFRQSLRLEQ------
FG05635.1 	CIKGSEKAVVEEVSVPLSEFRKNFP---------
AN7044.1  	ETQTPVDSRFPRTVVKSISMEEVKKMAFWQTN--
          	  .   .: .    *   .:.         :.
```
